# Trimodal prediction of speaking and listening willingness to help improve turn-changing modeling

**DOI:** 10.3389/fpsyg.2022.774547

**Published:** 2022-10-18

**Authors:** Ryo Ishii, Xutong Ren, Michal Muszynski, Louis-Philippe Morency

**Affiliations:** ^1^Language Technology Institute, Carnegie Mellon University, Pittsburgh, PA, United States; ^2^NTT Human Informatics Laboratories, NTT Corporation, Kanagawa, Japan

**Keywords:** speaking and listening willingness, willingness prediction, turn-taking, multi-task learning, multimodal signal processing

## Abstract

Participants in a conversation must carefully monitor the turn-management (speaking and listening) willingness of other conversational partners and adjust their turn-changing behaviors accordingly to have smooth conversation. Many studies have focused on developing actual turn-changing (i.e., next speaker or end-of-turn) models that can predict whether turn-keeping or turn-changing will occur. Participants' verbal and non-verbal behaviors have been used as input features for predictive models. To the best of our knowledge, these studies only model the relationship between participant behavior and turn-changing. Thus, there is no model that takes into account participants' willingness to acquire a turn (turn-management willingness). In this paper, we address the challenge of building such models to predict the willingness of both speakers and listeners. Firstly, we find that dissonance exists between willingness and actual turn-changing. Secondly, we propose predictive models that are based on trimodal inputs, including acoustic, linguistic, and visual cues distilled from conversations. Additionally, we study the impact of modeling willingness to help improve the task of turn-changing prediction. To do so, we introduce a dyadic conversation corpus with annotated scores of speaker/listener turn-management willingness. Our results show that using all three modalities (i.e., acoustic, linguistic, and visual cues) of the speaker and listener is critically important for predicting turn-management willingness. Furthermore, explicitly adding willingness as a prediction task improves the performance of turn-changing prediction. Moreover, turn-management willingness prediction becomes more accurate when this joint prediction of turn-management willingness and turn-changing is performed by using multi-task learning techniques.

## 1. Introduction

Turn-changing is an important aspect of smooth conversation, where the roles of speaker and listener change during conversation. For smooth turn-changing, conversation participants must carefully monitor the speaking and listening (turn-management) willingness of other conversational partners and consider whether to speak or yield on the basis of their own willingness and that of their partner.

To realize a dialogue system that can interact as smoothly as humans do, the dialogue system must be able to switch between listening and speaking at appropriate times, just as humans do. Therefore, predicting turn-changing can be helpful for conversational agents or robots as they need to know when to speak and take turns at the appropriate time. The field of human-computer interaction has long been dedicated to computational modeling of turn-changing. Many studies have focused on developing actual turn-changing (i.e., next speaker or end-of-turn) models that can predict whether turn-keeping or turn-changing will happen using participants' verbal and non-verbal behaviors (Ferrer et al., 2002; Schlangen, 2006; Chen and Harper, 2009; de Kok and Heylen, 2009; Laskowski et al., 2011; Kawahara et al., 2012; Jokinen et al., 2013; Holler and Kendrick, 2015; Ishii et al., 2015a,b, 2016a,b, 2017, 2019; Lammertink et al., 2015; Levinson, 2016; Hömke et al., 2017; Hara et al., 2018; Holler et al., 2018; Lala et al., 2018; Masumura et al., 2018, 2019; Roddy et al., 2018; Ward et al., 2018). These studies predicted turn-changing on the basis of verbal and non-verbal behaviors. However, the speaker and listener make the next speaking behavior based on their own willingness to speak or to listen to the partner's speaking. In addition to an individual's own willingness to speak, the decision regarding who takes the next turn to speak is also dependent on the willingness of the other. We believe that in order to predict the turn-changing, we should focus not only on the actions of the speaker and listener, but also on their willingness to speak, and by predicting these simultaneously, there is possibility to predict the turn-changing with higher accuracy.

**Figure 1:**
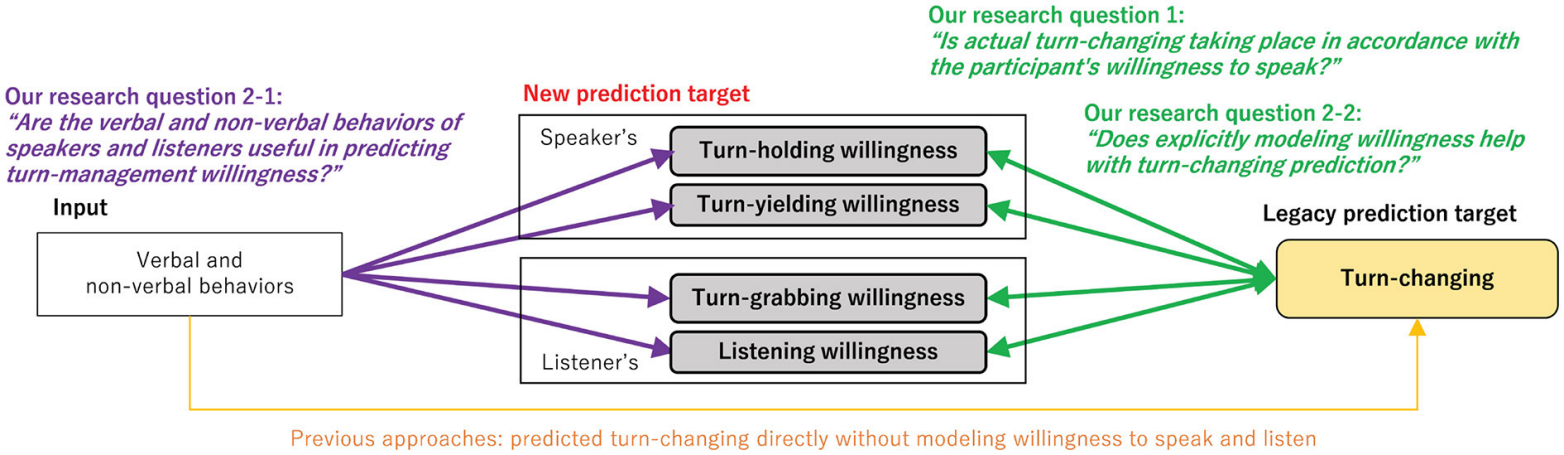
Overview of our research questions.

In this paper, we explore turn-management willingness during dyadic interactions with the goal of incorporating the modeling of willingness into a computational model of turn-changing prediction (see Figure 1). In this work, we study four types of willingness for speakers and listeners: turn-holding (speaker's willingness to speak), turn-yielding (speaker's willingness to listen), turn-grabbing (listener's willingness to speak), and listening (listener's willingness to listen). We also address two new research questions:

**Q1) Is actual turn-changing taking place in accordance with the participant's willingness to speak? In other words, is there consonance between turn-management willingness and actual turn-changing?**
**Q2-1) Are the verbal and non-verbal behaviors of speakers and listeners useful in predicting turn-management willingness?**
**Q2-2) Does explicitly modeling willingness help with turn-changing prediction?**

First, we analyze the relationship between turn-management willingness and actual turn-changing in our empirical study to address Q1. In particular, we investigate the relationship between the actual turn-changing and turn-management willingness of conversation participants to determine whether turn-changing occurs in accordance with the turn-management willingness in dialogue.

Second, we study the relative behavioral usefulness of features obtained from acoustic, linguistic, and visual modalities, from both speakers and listeners, to address Q2-1. Predicting this willingness directly has the potential to support conversational agents and robots with appropriate starting and ending utterances to have smooth conversations. To respond to Q2-2, we built predictive models for actual turn-changing prediction. As a first step, we use trimodal inputs, i.e., acoustic, linguistic, and visual cues, to directly predict turn-changing. As a second step, we incorporate willingness prediction into turn-changing prediction. This joint modeling approach is motivated by the intuition that humans are likely to control actual turn-changing on the basis of speaking and listening willingness. We build a multi-task model for joint turn-changing and willingness prediction, and then we evaluate the effectiveness of our proposed approach in terms of performance improvement. To answer these research questions, we collected a new dyadic dialogue corpus including audio and video recordings, and we transcribed the content of dyad conversations as well as acquired annotations of speaking and listening willingness scores for both conversation participants. The dataset was collected to empirically study the various combinations of speaking and listening willingness with turn-changing-based multimodal behavioral markers extracted from these three modalities (i.e., acoustic, linguistic, and visual). As the method for collecting willingness scores, it is very difficult for participants to evaluate their own internal state in dialogue. We deal with scores of willingness as perceived by several third parties observing the participants.

In Section 2, we review relevant related work and highlight our motivation to propose new approaches to turn-management willingness. Section 3 describes the corpus data collection using dyad interactions. Section 4 describes our analysis of turn-management willingness and actual turn-changing to address Q1. Sections 5 and 6 describe the implementation and evaluation of the proposed predictive models for turn-willingness and turn-changing to address Q2-1 and Q2-2. Section 7 discusses the results for Q1, Q2-1, and Q2-2. We conclude in Section 8 with a brief summary and mention of our future work.

## 2. Related work

### 2.1. Turn-changing and behaviors

Smooth turn-changing is an important aspect of social communication. It is one of the main means of coordinating one's communicative actions and interacting in a successful manner with others. In general, a turn refers to an interlocutors' right to speak, or “hold the floor,” while the partner (or partners) listens (Kendon, 1967). A turn typically consists of a stretch of speech that has a particular meaning or a message that the speaker intends to send across. Research on the elucidation of the mechanism behind turn-changing mainly began in the field of sociolinguistics. Sacks and colleagues (Sacks et al., 1974) proposed a turn-changing model, arguing that speaker switching occurs only at transition-related points (TRPs). Studies have demonstrated that verbal and non-verbal cues are important to indicate the presence or absence of turn-changing in dyad conversations (Lammertink et al., 2015; Levinson, 2016). Several studies have recently examined that non-verbal cues of conversation partners are discriminative for turn-changing prediction. In particular, it has been shown that eye-gaze behavior (Kawahara et al., 2012; Jokinen et al., 2013; Holler and Kendrick, 2015; Ishii et al., 2016a), head movement (Ishii et al., 2015b, 2017), respiration (Ishii et al., 2016b), and hand gestures (Holler et al., 2018) are strongly related to turn-changing.

To elaborate, the mutual interaction of gaze behavior is thought to contribute to smooth turn-changing (Kawahara et al., 2012; Jokinen et al., 2013; Holler and Kendrick, 2015; Ishii et al., 2016a). During turn-changing, the speaker tends to look at a listener in order to yield their turn to the listener. The listener who is to become the next speaker tends to look at the speaker, resulting in mutual gaze. During turn-keeping, listeners tend not to engage in mutual gazing with the speaker. Ishii et al. (2015b, 2017) have shown that nodding and head movements are more frequent during turn-changing. It has also been shown that the head movements of the speaker and listener tend to occur simultaneously during turn-changing. Ishii et al. (2014, 2016b) have also shown that participants breathe differently between turn-keeping and turn-changing. In detail, the speaker periodically takes quick breaths between utterances during turn-keeping. The speaker exhales and does not immediately begin taking a breath during turn-changing. The listener takes a larger-than-normal breath to start speaking during turn-changing. It has been reported that speaker's hand gestures tend to occur more during turn-keeping than during turn-changing (Holler et al., 2018).

### 2.2. Turn-changing prediction technology

As a result of previous research on conversation turns and behaviors, many studies have developed models for predicting actual turn-changing, i.e., whether turn-changing or turn-keeping will take place, on the basis of acoustic features (Ferrer et al., 2002; Schlangen, 2006; Chen and Harper, 2009; de Kok and Heylen, 2009; Huang et al., 2011; Laskowski et al., 2011; Eyben et al., 2013; Jokinen et al., 2013; Hara et al., 2018; Lala et al., 2018; Masumura et al., 2018, 2019; Roddy et al., 2018; Ward et al., 2018). They have used representative acoustic features from the speaker's speech such as log-mel and mel-frequency cepstral coefficients (MFCCs) as feature values.

Others have used linguistic features, such as BERT (Devlin et al., 2019), extracted from speaker's utterance text (Lala et al., 2018; Masumura et al., 2018, 2019; Roddy et al., 2018). Others have used visual features, such as overall physical motion (Chen and Harper, 2009; de Kok and Heylen, 2009; Dielmann et al., 2010; Roddy et al., 2018) near the end of a speaker's utterances or during multiple utterances. Moreover, some research has focused on detailed non-verbal behaviors, such as eye-gaze behavior (Chen and Harper, 2009; de Kok and Heylen, 2009; Huang et al., 2011; Jokinen et al., 2013; Ishii et al., 2015a, 2016a), head movement (Huang et al., 2011; Ishii et al., 2015b, 2017), mouth movement (Ishii et al., 2019), and respiration (Ishii et al., 2015a, 2016b). Specifically, information on the length of time and patterns of a speaker's gaze direction toward a listener during speaking, the amount of head movement, the patterns of the mouth opening and closing, and the amount of inspiratory volume are used as features for prediction.

However, many studies on turn-changing prediction use mainly features as mentioned above extracted from only speakers (Chen and Harper, 2009; de Kok and Heylen, 2009; Dielmann et al., 2010; Huang et al., 2011; Jokinen et al., 2013; Lala et al., 2018; Masumura et al., 2018, 2019; Roddy et al., 2018). Several studies have used limited features and modalities of listeners, such as linguistic, eye-gaze behavior, head movement, mouse movement, and respiration as mentioned above (Ishii et al., 2015a,b, 2016a,b, 2017, 2019; Masumura et al., 2018).

Therefore, previous studies (Ferrer et al., 2002; Schlangen, 2006; Chen and Harper, 2009; de Kok and Heylen, 2009; Laskowski et al., 2011; Kawahara et al., 2012; Jokinen et al., 2013; Holler and Kendrick, 2015; Ishii et al., 2015a,b, 2016a,b, 2017, 2019; Lammertink et al., 2015; Levinson, 2016; Hömke et al., 2017; Hara et al., 2018; Holler et al., 2018; Lala et al., 2018; Masumura et al., 2018, 2019; Roddy et al., 2018; Ward et al., 2018) have predicted the turn-changing based on the verbal and nonverbal behaviors of the speaker and listener. In other words, the focus has been on predicting the next behavior from the current behavior.

However, the speaker and listener make the next speaking behavior based on their own willingness to speak or to listen to the partner's speaking. In addition to an individual's own willingness to speak, the decision regarding who takes the next spoken turn is also dependent on the willingness of the other.

For example, even if the speaker is willing to continue speaking and performs verbal and non-verbal actions to start speaking, and the listener has a stronger willingness to speak, the speaker may not choose to continue speaking and may give up speaking to the listener. We believe that we should investigate not only on the actions of the speaker and listener, but also on their willingness to speak in order to predict the turn-changing. As a result, joint predictions might lead to improvement of the turn-changing performance.

To the best of our knowledge, our paper is the first to study the prediction of turn-management willingness in dyad interactions and the first attempt to explicitly add the willingness prediction task to the turn-changing predictive model. Furthermore, there is no prior research that investigates all acoustic, linguistic, and visual modalities of speakers and listeners for turn-changing prediction. Our study is the first to build a model for predicting willingness and turn-changing using trimodal information, including acoustic, linguistic, and visual cues of both speakers and listeners.

### 2.3. Human-agent interaction with turn-changing prediction

In the literature, researchers have mainly attempted to ensure smooth turn-changing, where the agent waits for its turn. Previous research predicts when human speech is completely finished, and the agent starts speaking after the human has finished speaking. In addition, the agent continues to make a predetermined utterance (continue turn-keeping) regardless of whether or not the human with whom it is interacting wants to speak or not. For example, in Schlangen (2006) and Atterer et al. (2008), algorithms were developed to predict turn-endings as soon as possible such that the system can immediately respond in order to simulate human-like behavior. In Raux and Eskenazi (2008), the authors demonstrated how audio features are used to detect an end-of-turn as soon as possible; thus, an agent can start to speak as soon as possible. In human-agent interactions, an agent attempts to acquire a turn and starts uttering at an appropriate time by using the prediction of a turn-changing predictive model. In Jonsdottir et al. (2008) and Jonsdottir and Thórisson (2009), a real-time turn-changing model was developed to minimize the gaps of silence between the speech turns of a human and system.

Also, using our estimation of turn-management willingness, agents may be able to facilitate users' in speaking on the basis of the users' willingness. For example, although a listener may strongly want to take a turn, he/she may not actually be able to do so (i.e., the speaker does not yield to him/her). At such moments, the agent may be able to prompt the listener to start speaking using verbal and non-verbal behavior (the discrepancies between the turn-management willingness of speakers and listeners and actual turn-changing will be reported in Section 4).

## 3. New MM-TMW corpus

### 3.1. Dialogue data collection

We collected a new corpus (named the “MM-TMW Corpus”) that contains verbal and non-verbal behavioral information on human-human dialogues. It consists of 12 face-to-face conversations of people who had never met before (12 groups of 2 people). The participants were 24 Japanese (12 males and 12 females) in their 20–50 s (mean: 32.0, STD: 8.4). Participants were recruited from the general public at large through a staffing agency. The age difference between pairs and the number of male-female pairs were set to be as varied as possible. All participants gave informed consent. They were seated in close proximity to each other in a quiet environment. The conversations were structured to be about multiple topics, including taxes and social welfare balance. The lengths of these conversations were unified to be around 10 min. The total time of all conversations was 120 min. The participants' voices were recorded by a headset microphone. The entire discussions were recorded by a camera. We also took upper body videos of each participant recorded at 30 Hz. A professional transcribed all Japanese utterances, and another double-checked the transcripts.

### 3.2. Annotation of turn-management willingness

As a first step, professional annotators identified the spoken utterance segments using the annotation scheme of the inter-pausal unit (IPU) (Koiso et al., 1998). Each start and end of an utterance was denoted as an IPU. When a silence interval of 200 ms or more occurred, the utterance was separated. Therefore, if an utterance was produced after a silent period of less than 200 ms, it was determined to be a continuation of the same utterance.

When a silence interval of 200 ms or more occurred, the utterances were separated. Since this IPU can determine the start and end of an utterance using only the duration of silent segments, it is very convenient and useful when performing real-time utterance segment detection. For this reason, many studies on turn-changing have utilized these IPU units of utterances (Ferrer et al., 2002; Schlangen, 2006; Chen and Harper, 2009; de Kok and Heylen, 2009; Laskowski et al., 2011; Kawahara et al., 2012; Jokinen et al., 2013; Holler and Kendrick, 2015; Ishii et al., 2015a,b, 2016a,b, 2017, 2019; Lammertink et al., 2015; Levinson, 2016; Hömke et al., 2017; Hara et al., 2018; Holler et al., 2018; Lala et al., 2018; Masumura et al., 2018, 2019; Roddy et al., 2018; Ward et al., 2018). In Japanese, it is considered appropriate to adopt 200 ms as the threshold for an appropriate IPU to be segmented as a single utterance; if the threshold is set at around 100 ms, utterances with only words are extracted or are cut off by a brief pause in occurrence. If the time is set to about 300 ms, there is the problem of different utterances being connected as a single utterance. Therefore, a threshold of 200 ms has been used in many studies. The largest corpus of Japanese speech (Maekawa, 2003), which contains speech signals and transcriptions of about 7 million words along with various annotations like parts of speech and phonetic labels, also uses the 200-ms threshold as an appropriate threshold for segmenting Japanese speech. We took these considerations into account and adopted the 200-ms threshold.

**Figure 2:**
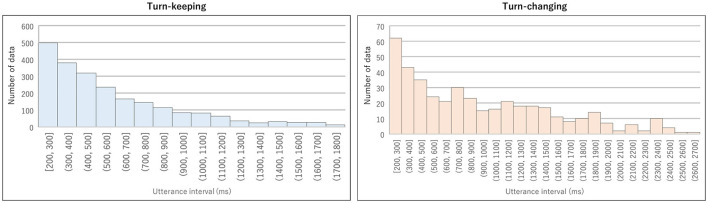
Histogram of utterance interval duration for turn-keeping (left) and turn-changing (right) from MM-TMW Corpus.

We excluded backchannels without specific vocal content from the extracted IPUs. Next, we considered IPU pairs produced by the same person in temporally adjacent IPU pairs as turn-keeping and those produced by different people as turn-changing. Specifically, data was excluded when the IPU pair utterance interval was less than 200 ms. The total number of pairs was 2,208 for turn-keeping and 442 for turn-changing. Histograms of the utterance interval duration for turn-keeping and turn-changing are shown in Figure 2. The average duration was about 577 ms for turn-keeping and 892 ms for turn-changing.

It is very difficult for participants to evaluate their own internal state in dialogue. Therefore, for the sake of accuracy and objectivity of assessment of the internal state, such as emotion and engagement, the annotation from a third party annotator who does not participate in the dialogue has been collected in many studies (Devillers et al., 2005; Busso et al., 2008; Reidsma and op den Akker, 2008; Huang et al., 2010; Nicolaou et al., 2011; Ishii et al., 2013; Kumano et al., 2015). The subject's internal state obtained in this way is not the subject's internal state but only the internal state as perceived by the observer. We collected turn-management scores from multiple external observers using the same annotation method for multiple external observers as a reference. Thus, we deal with the turn-management willingness of the dialogue participants as perceived by the observers.

**Figure 3:**
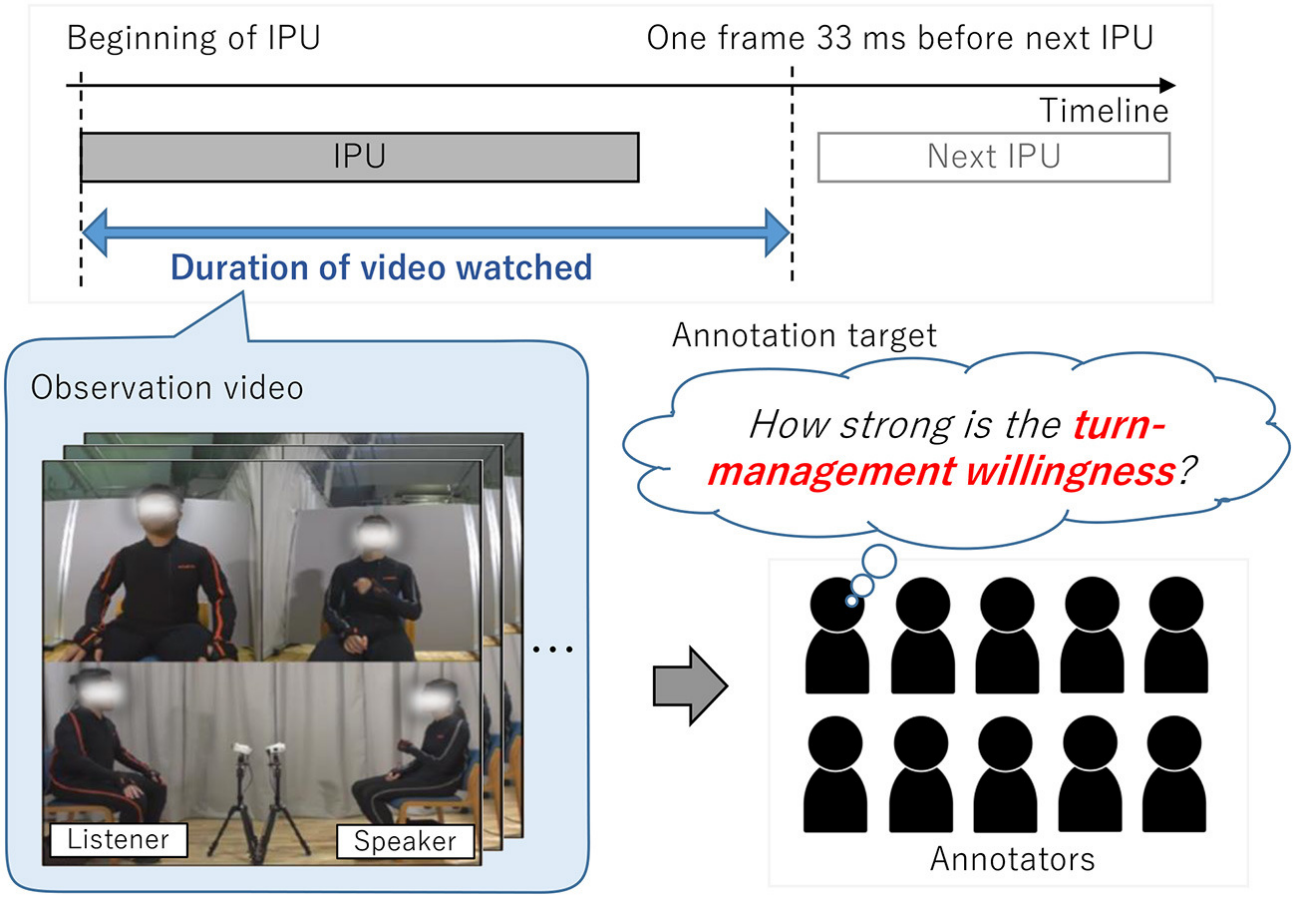
Scheme for annotating speaking and listening willingness of speaker and listener.

The 10 annotators carefully watched each video from the beginning of one utterance (IPU) to the point of one frame (i.e., 33 ms) before the beginning of the next utterance to annotate willingness scores (see Figure 3). The annotators were not aware of who would become the next speaker because they could only watch the video until the moment just before the start of the next speaker. This approach was taken to avoid affecting the annotators' judgement on the willingness of the speakers and listeners to speak and listen. For very short IPUs of less than 1 s, we set the start of the video to a moment earlier than the start time of the IPUs so that the annotators could view at least 1 s of video. In addition, the content of the current utterance and that of the past dialogue was considered to be important for judging turn-management willingness. Therefore, the annotators observed the utterances in order, starting with the first utterance at the beginning of the dialogue. They could refer to contextual information on past dialogue to annotate the willingness score. The annotation order for the 12 dialogues was randomized for each annotator. For each video, the annotators provided scores to four types of turn-management willingness of speakers and listeners:

- **Turn-holding willingness (speaker's willingness to speak)**: Does the speaker have the will to hold the turn (continue speaking)?
- **Turn-yielding willingness (speaker's willingness to listen)**: Does the speaker have the will to yield the turn (listen to listener's speaking)?
- **Turn-grabbing willingness (listener's willingness to speak)**: Does the listener have the will to take a turn (start speaking)?
- **Listening willingness (listener's willingness to listen)**: Does the listener have the will to continue listening to the speaker?

The annotators scored each willingness index on a 5-point Likert scale, where 1 meant “He/she is not showing willingness,” 5 meant “He/she is showing strong willingness,” and 3 meant “uncertain.” We had the 10 annotators score all videos to ensure good reliability. We calculated the rater agreement using the Intraclass Correlation Coefficient (ICC). The ICC scores for all four categories were over **0.870**: *ICC*(2, 10) = **0.904** for speaker's willingness to speak, *ICC*(2, 10) = **0.877** for speaker's willingness to listen, *ICC*(2, 10) = **0.878** for listener's willingness to speak, and *ICC*(2, 10) = **0.875** for listener's willingness to listen. A high annotation agreement measured by ICC suggests that the data was very reliable. We used the average values of the 10 annotators as willingness scores.

## 4. Analysis of turn-management willingness and turn-changing patterns (related to Q1 research question)

### 4.1. Overall trends

**Figure 4:**
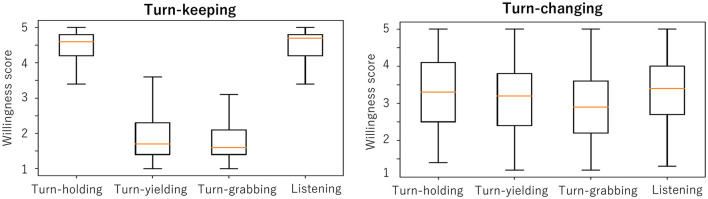
Average willingness scores in turn-keeping (left) and turn-changing (right).

In this section, we analyze the relationship between willingness scores and actual turn-changing or turn-keeping as an empirical study. Figure 4 shows box plots of each willingness score in our corpus, separated between turn-keeping and turn-changing to investigate the overall relationship between them. When turn-keeping happened, the average scores for the speaker's speaking willingness and listener's listening willingness were higher than 4.5, which is very high. In contrast, the average scores for the speaker's listening willingness and listener's speaking willingness were less than 2.0, which is very low.

This means that the person who becomes the next speaker has a high speaking willingness and the person who becomes the next listener has a high listening willingness in turn-keeping. In other words, “*the speaker prefers to continue speaking, and the listener prefers to continue listening”* during turn-keeping. Corresponding *t*-tests were conducted to determine if there was a statistically significant difference between turn-holding and turn-yielding willingness scores. The results showed that there was a significant difference between turn-holding and turn-yielding scores [*t*_(2, 219)_ = 3.70, *p* < 0.001]. Similarly, there was a significant difference between scores for turn-grabbing and listening willingness [*t*_(2, 219)_ = 3.56, *p* < 0.001]. This result is not too surprising, and it suggests that the participants' willingness and turn-keeping were consistent (i.e., there was no dissonance).

For turn-changing, all the average willingness scores were from 3.0 to 3.5, with larger standard deviations. This result suggests that around turn-changing, the person who becomes the next speaker does not always have a high speaking willingness, and the person who becomes the next listener does not always have a high listening willingness. The results of a corresponding *t*-test showed that there was no significant difference between turn-holding and turn-yielding scores and between turn-grabbing and listening willingness scores.

Therefore, the relationship between willingness and turn-changing is more complex than expected. There may be cases of dissonance between willingness and actual turn-changing as answer to research question Q1.

### 4.2. Detailed analysis of combinatorial patterns

To study this relationship in detail, we analyzed the relative ordering of speaking and listening willingness scores when co-occurring with turn-keeping or turn-changing. We denote willingness combinatorial patterns as (*r*_*SS*_, *r*_*SL*_, *r*_*LS*_, *r*_*LL*_) to represent the ordering position among all willingness scores, where *r*_*SS*_, *r*_*SL*_, *r*_*LS*_, *r*_*LL*_ are the ranks of the speaker's speaking, speaker's listening, listener's speaking, and listener's listening willingness, respectively. For example, *r*_*SS*_ = 1 if the speaker's speaking willingness score has the highest value among all of the scores. As another example, when the scores for speaker's speaking, speaker's listening, listener's speaking, and listener's listening willingness were 3.57, 2.95, 1.82, and 4.44, the corresponding pattern was denoted as (2, 3, 4, 1).

**Figure 5:**
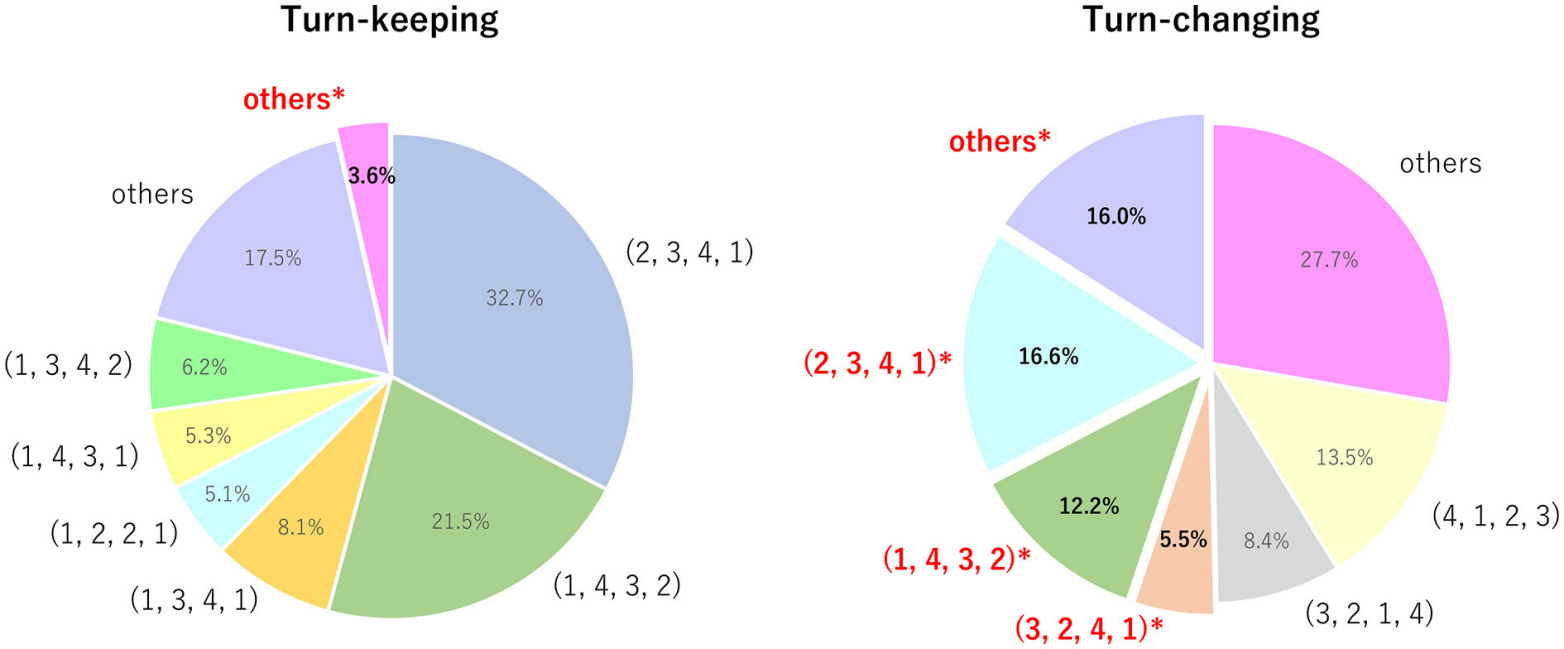
Frequency of willingness combination patterns for turn-keeping (left) and turn-changing (right). (*r*_*SS*_, *r*_*SL*_, *r*_*LS*_, *r*_*LL*_) indicates ranks of speaker's and listener's speaking and listening willingness, where highest value was ranked as 1. “others” includes patterns taking up less than 5%. Cases of conflict are marked with ^*^, which means that actual turn-changing/keeping was against participants' willingness. Detailed analysis is given in Section 4.

Figure 5 shows the frequency of the willingness combinatorial patterns for turn-keeping and turn-changing. In this figure, “others” includes patterns taking less than 5% for turn-keeping or turn-changing. We define consonance as moments when actual turn-changing/keeping matched the participants' willingness. We also define dissonance as moments when actual turn-changing/keeping went against their willingness, and these are marked with ^*^. We discuss these results in the following paragraphs.

#### 4.2.1. Consonance in combinatorial patterns

Consonance cases happen during turn-keeping when “*the speaker prefers to continue speaking”* (*r*_*SS*_<*r*_*SL*_) or “*the listener prefers to continue listening”* (*r*_*LS*_>*r*_*LL*_). As shown in Figure 5, consonance patterns took up **96.4%** for turn-keeping, which suggests that there were few cases of dissonance between willingness and turn-keeping. For turn-changing, consonance cases happen when “*the speaker prefers to start listening”* (*r*_*SS*_<*r*_*SL*_) or “*the listener prefers to start speaking”* (*r*_*LS*_<*r*_*LL*_). Consonance patterns took up **49.6%** for turn-changing. This means that consonance cases almost always happen during turn-keeping but not during turn-changing.

#### 4.2.2. Dissonance in combinatorial patterns

During turn-keeping, dissonance cases happen when “*the speaker wants to listen, and the listener wants to speak”* (*r*_*SS*_>*r*_*SL*_ and *r*_*LS*_<*r*_*LL*_). This means that the preferences of both the speaker and listener are in harmony. Thus, a turn-change should occur. For turn-changing, dissonance cases happen when “*the speaker wants to speak, and the listener wants to listen”* (*r*_*SS*_<*r*_*SL*_ and *r*_*LS*_>*r*_*LL*_). In this case, both participants want to keep their role. In Figure 5, we marked all dissonance cases with ^*^. As shown in the figure, **3.6%** of cases of dissonance occurred for turn-keeping, while **50.4%** were for turn-changing. The large number of cases between willingness scores and turn-changing indicates the importance of estimating the willingness score. The next speaker decided against his or her own willingness as well as their partner's willingness. Such occurrences have the potential to frustrate participants during a conversation.

## 5. Turn-management willingness and turn-changing predictive models (related to Q2-1 and Q2-2)

### 5.1. Motivation

The analysis results in Section 4 suggest that the willingness scores sometimes had discrepancies with actual turn-changing. The accuracy could be further improved by performing multi-task learning on willingness and turn-changing since they have a strong relationship. Multi-task learning is the process of learning latent representations that are shared among multiple related tasks. In the field of deep learning, deep learning-based approaches in which the parameters of hidden layers are shared by multiple tasks are well-established and commonly used. Learning multiple related tasks simultaneously has been demonstrated to improve the prediction performance of each single task while exploiting commonalities and differences across tasks (Ruder, 2017). For that reason, we hypothesize that joint prediction of turn-management willingness and turn-changing could lead to improved performance on each task in comparison with training two predictive models separately.

To address **Q2-1**, we implemented three kinds of models for predicting turn-management willingness by using the multimodal behaviors of either the speaker or listener or both of them. By investigating and comparing the performance of the models, we demonstrate that turn-management willingness can be predicted by using the multimodal behaviors of speakers and listeners. To address **Q2-2**, we also implemented models for predicting turn-changing that jointly predict turn-management willingness on the basis of single turn-management predictive models. By comparing the performance of the models between using multi-task learning and single-task learning, we demonstrate that joint-willingness prediction can improve the performance of turn-changing prediction.

### 5.2. Multimodal features

We used the features of behaviors extracted during IPUs (i.e., the time between the start and end of an IPU) as input for the predictive models the same as other research on turn-changing prediction (Ferrer et al., 2002; Schlangen, 2006; Chen and Harper, 2009; de Kok and Heylen, 2009; Laskowski et al., 2011; Kawahara et al., 2012; Jokinen et al., 2013; Holler and Kendrick, 2015; Lammertink et al., 2015; Levinson, 2016; Hömke et al., 2017; Holler et al., 2018; Lala et al., 2018; Masumura et al., 2018). This means that our models could predict willingness and turn-changing at the end of a speaker's utterance (IPU). Since the duration between the end of one speaker's utterance and the start of the next speaker's utterance is about 629 ms on average, our models could predict willingness and turn-changing about 629 ms before actual turn-keeping and turn-changing happen.

Our goal is not necessarily to propose the most complex multimodal fusion approach but rather to study willingness and its impact on turn-changing precision. Recently, high-level abstract features, which are extracted from large-scale pre-trained neural network models, have been very useful for many various prediction tasks.

In deep learning, a large-scale pre-trained model is a model that has been trained on a large dataset. In general, generic features common to a specific domain task (e.g., image classification, text prediction, speech recognition, etc.) can be trained in a hidden layer and extracted for transition learning (Han et al., 2021). Such pre-trained models can be used to extract high-level abstract audio, visual, and written features even in stimuli not used for training. Such high-abstracted features are known to be more useful in various estimation tasks than speech, image, and text features of interpretable features, which are hand-crafted and designed on the basis of prior domain knowledge.

For example, in one of the most recent pieces of research (Soleymani et al., 2019), a model was proposed to estimate self-disclosure utterances using the multimodal features of acoustic, linguistic, and visual modalities while utterances take place. It was demonstrated that the latest high-level abstract features, such as those of VGGish (Hershey et al., 2017), BERT (Devlin et al., 2019), and ResNet-50 (He et al., 2016), were more useful than interpretable features hand-crafted and designed on the basis of prior domain knowledge, such as mel-frequency cepstral coefficients that represent the power spectrum of a sound similar to that approximated in the human auditory system (MFCCs) (Eyben et al., 2013), LIWC, which analyzes text to identify the psychological categories of words (Kahn et al., 2007), and facial action units, which can be analyzed to identify facial expressions (AU) (Baltrusaitis et al., 2018).

These are human-empirically designed features that extract only a limited subset of the features of speech, language, and video. On the other hand, pre-trained models using neural networks with large datasets extract features from a wide variety of hidden aspects that cannot be designed by humans.

To implement willingness prediction models, we used automatically extracted high-level abstract features from the recorded acoustic, linguistic, and visual modalities on the basis of an existing study (Soleymani et al., 2019) as mentioned above.

#### Acoustic Modality

We used VGGish (Hershey et al., 2017), which is a pre-trained deep convolutional neural network, to extract features of the acoustic modality from audio data. VGGish is a variant of the VGG model (Simonyan and Zisserman, 2015), trained on a large YouTube dataset to classify an ontology of 632 different audio event categories (Gemmeke et al., 2017), involving human sounds, animal sounds, natural sounds, etc. The audio files were converted into stabilized log-mel spectrograms and fed into the VGG model to perform audio classification. The output 128-dimensional embeddings were post-processed by applying a PCA transformation (that performs both PCA and whitening). Therefore, each audio sample was encoded as a feature with a shape of *T*×128, where *T* is the number of frames. During natural conversations, listeners are not always absolutely silent; there are short backchannel responses or echoes of what speakers have said. Therefore, the VGGish features could be extracted from the listeners' acoustic signals in addition to speakers' acoustic signals.

#### Linguistic modality

We applied a data-driven approach (BERT) (Devlin et al., 2019) to extract linguistic representations. BERT is a multi-layer bidirectional Transformer network that encodes a linguistic sequence into a fixed-length representation. We used a pre-trained BERT model on Japanese Wikipedia^1^ to transfer each utterance into a 768-dimensional feature. The BERT feature could be extracted from the listeners' speech in addition to speakers' speech similarly to acoustic features since listeners often have short backchannel responses.

#### Visual modality

For visual information, high-level representations were extracted using ResNet-50 (He et al., 2016), which is a deep residual convolutional neural network for image classification. We used a ResNet-50 model that was trained on ILSVRC2012 (Russakovsky et al., 2015), a large scale dataset that contains about 1.2 million training samples in 1,000 categories, to provide good generalization and yield robust features. The feature vector for a video sequence consisted of a 2, 048-dimensional vector obtained from the penultimate layer for each frame. As a result, the extracted feature was in the shape of *T*×2048.

### 5.3. Predictive models

**Figure 6:**
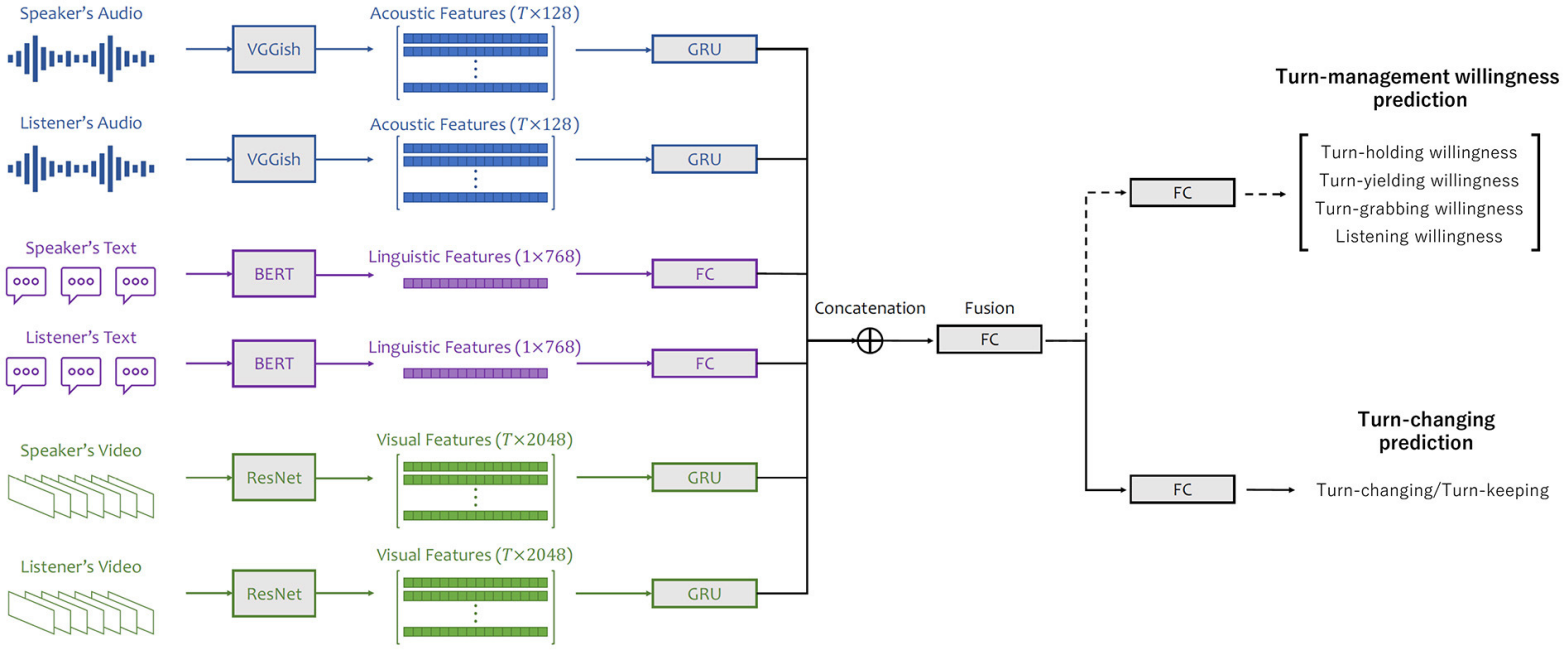
Architecture of multi-task model with input features of acoustic, linguistic, and visual modalities from speaker and listener.

Turn-management willingness and turn-changing were first predicted individually using regression models (for predicting turn-management willingness scores) and classification models (for turn-changing/keeping prediction), respectively. A multi-task model was then learned to jointly predict willingness and turn-changing/keeping. This helps to understand the impact of modeling willingness on turn-changing explicitly. The architecture of our model is illustrated in Figure 6.

#### Turn-management willingness prediction

We formulated turn-management willingness prediction as a regression task and average willingness scores from the 10 annotators as the ground truth. We trained deep neural network-based predictive models to estimate willingness scores. The unimodal features were first fed into individual processing modules to be further processed as 64-dimensional embeddings. For acoustic and visual modalities, the processing module was a one-hidden-layer gated recurrent unit (GRU) (Cho et al., 2014). A fully connected (FC) layer was used for the linguistic modality. The embeddings were then concatenated together and forwarded into a FC layer with an output size of 192 for fusion. A final linear layer followed, outputting four predicted willingness scores. We used mean squared error (MSE) as our loss function.

#### Turn-changing prediction

Turn-changing prediction was considered a classification task. Each turn was labeled as either turn-changing or turn-keeping, depending on whether the current listener became the next actual speaker. The classification model followed the same structure as the regression one, except that it output a two-dimensional vector for prediction. Cross entropy (CE) was used as the loss function.

#### Multi-task prediction (joint prediction of turn-management willingness and turn-changing)

To embed knowledge on willingness into turn prediction, our proposed multi-task model jointly predicts willingness scores and turn-changing/keeping. In particular, the turn-management willingness and turn-changing prediction tasks are simultaneously learned by the proposed multi-task predictive model. The architecture of the proposed model is designed on the basis of the neural architecture discussed above. The main difference is that, after the fusion layer, there is a FC layer for each task. The entire loss function is a weighted average of MSE and CE with weights of 1 and 2, respectively.

## 6. Experiments with predictive model (related to Q2-1 and Q2-2)

### 6.1. Experimental methodology

To answer **Q2-1**, we implemented different predictive models for turn-management willingness prediction based on the multimodal behaviors of either the speaker or listener or both. We investigated and compared the performance of the models to demonstrate that turn-management willingness can be predicted by using the multimodal behaviors of speakers and listeners. To answer **Q2-2**, we also implemented models for turn-changing prediction that jointly predict turn-management willingness and turn-changing. We compared the performance of the multi-task learning models and single-task models to demonstrate that incorporating willingness into turn-changing predictive models improves turn-changing prediction.

All models were trained using the Adam (Kingma and Ba, 2015) optimizer with a learning rate of 0.0001 for 50 epochs. The batch size was 64. Furthermore, we added dropout layers with a rate of 0.1 for the FC layers. Leave-one-dyad-out testing (12-fold cross-validation method) was used to evaluate model performance. With the testing, we evaluated how much the willingness and turn-changing of new dyads can be predicted.

For the willingness prediction task, we report the concordance correlation coefficients (CCCs) between predicted and actual scores (i.e., annotated ground truth). A high CCC value indicates high agreement between the values of the predicted scores and ground truth. This means that prediction and ground truth values are similar to each other, and the general trend changes for both signals are the same (Muszynski et al., 2019). We compared the predictions of pairs of regression models by means of two-sided Wilcoxon signed rank tests at a 0.05 significance level (Wilcoxon, 1945). For the classification task, we evaluated the performance using F1 scores weighted by the label proportion since the numbers of turn-changing and turn-keeping labels were imbalanced in our dataset. The predictions of pairs of classifiers were made by means of a McNemar test at a 0.05 significance level (McNemar, 1947).

### 6.2. Results

#### 6.2.1. Comparison method of predictive models

Models were built using combinations of different input features. The results for willingness and turn prediction are shown in Table 1. Model (1) was the base model of prediction. It was a random predictive model that randomly generates scores and classes from learning data without using the features of speakers and listeners. The CCCs of the willingness prediction for model (1) were –0.011 for turn-holding, –0.013 for turn-yielding, –0.025 for turn-grabbing, and 0.007 for listening. The F1 score for turn-changing prediction was 0.528.

**Table 1:** Results of turn-management willingness and turn-changing prediction.

	**Features**							**Willingness prediction (CCC)**				**Turn-changing**

Model	**Speaker**			**Listener**			**MtL**	**Speaker**		**Listener**		**Prediction**
#	**Acoustic**	**Ling**.	**Visual**	**Acoustic**	**Ling**.	**Visual**		**Turn-holding**	**Turn-yielding**	**Turn-grabbing**	**Listening**	**(F1 Score)**
(1)								–0.011	–0.013	–0.025	0.007	0.528
(2)	×							0.399	0.306	0.167	0.241	0.703
(3)		×						0.371	0.360	0.295	0.296	**0.735** ^(5)*^
(4)			×					0.182	0.127	0.146	0.135	0.678
(5)				×				**0.413** ^(2)*^	**0.397** ^(2)**^	**0.506** ^(6)**^	**0.478** ^(3)**^	0.715
(6)					×			0.190	0.215	0.318	0.291	0.709
(7)						×		0.069	0.074	0.099	0.095	0.660
(8)	×			×				**0.513** ^(2)**, (5)**, (9)**^	**0.460** ^(2)**, (5)**, (9)**^	**0.532** ^(2)**, (5)**, (9)**^	**0.513** ^(2)**, (5)**, (9)**^	0.720^(2)*^
(9)		×			×			0.411^(3)**, (6)**^	0.409^(3)**, (6)**^	0.434^(3)**, (6)**^	0.403^(3)**, (6)**^	**0.745** ^(6)*^
(10)			×			×		0.201^(4)*, (7)**^	0.152^(4)*, (7)**^	0.149^(7)**^	0.164^(4)*, (7)**^	0.675^(4)**, (7)**^
(11)	×	×	×					0.497^(12)**^	0.425^(12)**^	0.344	0.395	0.759
(12)				×	×	×		0.412	0.376	0.485^(11)**^	0.465^(11)**^	0.711
(13)	×	×	×	×	×	×		**0.556** ^(5)**, (11)**^	**0.504** ^(5)**, (11)**^	**0.573** ^(5)**, (12)**^	**0.549** ^(5)**, (12)**^	**0.771** ^(3)**, (11)*, (12)**^
(14)	×	×	×	×	×	×	×	**0.580** ^(8)**, (13)*^	**0.530** ^(8)**, (13)*^	0.572^(8)**^	**0.560** ^(8)**, (13)*^	**0.797** ^(9)**, (13)**^

Each row represents results of model with different configuration of input features. Section (6) describes experiments in detail. CCC is reported for each model for turn-management willingness prediction. F1 score is reported for turn-changing prediction. Results of running two-sided Wilcoxon signed rank among models (2) to (7), (8) to (10), and (11) to (13) are shown. The highest performance per a given configuration of input features is marked in bold. Results are shown for pairs of two conditions, (2) and (5) vs. (8), (3) and (6) vs. (9), (4) and (7) vs. (10) and (3) vs. (13), and (8) vs. (13) for willingness and turn-changing prediction, and (13) vs. (14) for willingness and turn-changing prediction. * stands for p < 0.05, while ** stands for p ≪ 0.001.

Models (2) to (7) were predictive models fed with acoustic, linguistic, or visual modalities of speaker or listeners, respectively. The performance of these models was evaluated to verify if each modality was effective and discriminative for our prediction tasks. Models (8) to (10) were predictive models that were fed with a single modality of speakers and listeners. In detail, model (8) was applied to speech features, while model (9) was fed with linguistic features. Furthermore, model (10) was trained on visual features. By comparing models (8) to (10) with models (2) to (7), we could verify whether using the features of speakers and listeners is more effective than using the features of either speaker or listener. Models (11) and (12) were predictive models that used features from the three modalities of speakers or listeners. Model (13) was a predictive model that learns on input features extracted from the three modalities of both the speaker and the listener. By comparing models (11) to (13) with models (2) to (7), we could examine the usefulness and discriminative power of multimodal features over features extracted from a single modality. These comparisons were made to address Q2-1. Model (14) was a multi-task learning predictive model trained on the three modalities of speakers and listeners to simultaneously predict turn-management willingness and turn-changing. This model (14) was compared with model (13), which does not use multi-task learning techniques. These comparisons were intended to respond to Q2-2.

#### 6.2.2. Results of turn-management willingness prediction using speaker/listener behaviors (related to Q2-1)

First, we examined which of the speaker's and listener's unimodal features was most useful for estimating turn-management willingness when used alone. As shown in Table 1, models (2) to (7) used only the unimodal features of the speaker or listener independently. Comparing the performances of the models among (2) to (7), the CCCs for turn-holding, turn-yielding, turn-grabbing, and listening prediction for model (5), 0.413, 0.397, 0.506, and 0.478, were significantly higher than those of the models that had the second highest values, 0.399 for (2), 0.360 for (3), 0.318 for (6), and 0.296 for (3) (*p* < 0.05 or *p* < 0.001). This suggests that a listener's acoustic features are most useful for predicting speaker and listener turn-management willingness among the unimodal features of speakers and listeners.

We examined whether using unimodal features from both speakers and listeners to estimate turn-management willingness is more useful than using those from one of them alone. As shown in Table 1, models (8) to (10) used only the unimodal features of both the speaker and listener independently. We compared model (8) with models (2) and (5) to demonstrate that acoustic features from both the speaker and listener are more useful than using only those from either the speaker or listener. The CCCs for model (8), 0.513, 0.460, 0.532, and 0.513, were significantly higher than those of models (2) and (5) (*p* < 0.001). We compared model (9) with models (3) and (6) to demonstrate that linguistic features from both the speaker and listener are more useful than using only those from either the speaker or listener. The CCCs for model (9), 0.411, 0.409, 0.434, and 0.403, were significantly higher than those of models (3) and (6) (*p* < 0.001). We compared model (10) with models (4) and (7) to demonstrate that linguistic features from both the speaker and listener are more useful than using only those from either the speaker or listener. The CCCs for model (8), 0.201, 0.152, 0.149, and 0.164, were significantly higher than those of models (4) and (7) except for the case of turn-grabbing willingness between models (4) and (10) (*p* < 0.05 of *p* < 0.001). These suggest that using unimodal features from both the speaker and listener to estimate turn-management willingness was more useful than using them alone.

Comparing models (8) to (10), the CCCs of turn-management willingness prediction for model (10) were significantly highest (*p* < 0.001). This suggests that acoustic features are most useful for turn-management prediction when using unimodal features from both the speaker and listener.

We examined which of the speaker's or listener's multimodal features were most useful for estimating turn-management willingness. As shown in Table 1, models (11), (12), and (13) used trimodal features of the speaker, listener, and both independently.

Comparing models (11) and (12), the CCCs of turn-holding and turn-yielding prediction for model (11), 0.497 and 0.425, were significantly higher than those of model (12), 412 and 0.376 (*p* < 0.001). In contrast, the CCCs of turn-grabbing and listening prediction for model (12), 0.485 and 0.465, were significantly higher than the CCCs for model (11), 0.344 and 0.395 (*p* < 0.001). This suggests that speaker/listener features are more useful for predicting speaker/listener turn-management willingness than listener/speaker willingness.

Comparing model (13) with models (11) and (12), model (13) with all features performed best, 0.556 for turn-holding, 0.504 for turn-yielding, 0.573 for turn-grabbing, and 0.549 for listening, being significantly higher than models with speaker features (11) or listener features (12) (*p* < 0.001). This suggests that a model using features from both speakers and listeners outperforms a model using them from one person. Comparing model (5), which had the highest CCC when using only unimodal features, and (13), which had the highest CCC when using multimodal features, the CCCs of turn-holding and turn-yielding prediction for model (13) were significantly higher than that of model (5) (*p* < 0.001). We found an overall improvement in turn-management willingness prediction by fusing the multiple features of speakers and listeners.

#### 6.2.3. Results of turn-changing prediction using speaker/listener behaviors

We implemented and evaluated the performance of turn-changing predictive models (2) to (13) similarly to the turn-management predictive models to assess the effect of multi-task learning on turn-changing prediction. We report the performance of the models to confirm whether our extracted speaker and listener features were useful for turn-changing prediction.

First, we examined which of the speaker's and listener's unimodal features was most useful for predicting turn-changing when used alone. Comparing the performances of the models among (2) to (7), the F1 score for model (3), 0.735, was significantly higher than that of the model that had the second highest values, 0.715 for (5) (*p* < 0.05). This suggests that a speaker's linguistic features are most useful for predicting turn-changing among the unimodal features of speakers and listeners.

We examined whether using unimodal features from both the speaker and listener to predict turn-changing is more useful than using them alone. We compared model (8) with models (2) and (5) to demonstrate that acoustic features from both the speaker and listener are more useful than using only those from either the speaker or listener. The F1 score for model (8), 0.720, was significantly higher than that of model (2) (*p* < 0.05). We compared model (9) with models (3) and (6) to demonstrate that linguistic features from both the speaker and listener are more useful than using only those from either the speaker or listener. The F1 score for model (9), 0.745, was significantly higher than that of model (6) (*p* < 0.05). We compared model (10) with models (4) and (7) to demonstrate that linguistic features from both the speaker and listener are more useful than using only those from either the speaker or listener. The F1 score for model (10), 0.675, was significantly higher than those of models (4) and (7) (*p* < 0.05 and *p* < 0.001). These suggest that using unimodal features from both the speaker and listener for estimating turn-changing was more useful than using only those from one of them.

Comparing models (8) to (10), the CCCs of turn-management willingness prediction for model (9) were significantly highest (*p* < 0.001). This suggests that acoustic features are most useful for turn-management prediction when using unimodal features from both the speaker and listener.

We examined which of the speaker's or listener's multimodal features were most useful for predicting turn-changing. As shown in Table 1, models (11), (12), and (13) used trimodal features of the speaker, listener, and both independently.

Comparing models (11) and (12), the F1 score of turn-changing prediction for model (11), 0.759, was significantly higher than that of model (12), 711 (*p* < 0.001). This suggests that the speaker's features are more useful for predicting turn-changing.

Comparing model (13) with (12), model (13) with all features performed best, 0.771, which was significantly higher than model (12) (*p* < 0.001). This suggests that the model using the features from speaker and listener more effective than the model only using the features of speaker or listener. We found an overall improvement in turn-changing prediction by fusing multiple speaker and listener features. These results are in line with previous research that similarly used both speaker and listener behaviors for turn-changing prediction (Ishii et al., 2016a,b, 2019).

The performance of our turn-changing predictive models was high [i.e., 0.771 for model (4)] even though the prediction task is known to be difficult and our dataset is relatively small. As an alternative, features from pre-training models such as VGGish, BERT, and ResNet-50 could be used to mitigate our relatively small dataset. Turn-changing predictive models (2) to (4) can serve as a baseline for evaluating the effect of using multi-task learning.

#### 6.2.4. Results of multi-task prediction of turn-management willingness and turn-changing (related to Q2-2)

We first analyzed whether applying multi-task learning to turn-management willingness and turn-changing prediction can improve turn-changing prediction. Model (14) used multi-task learning in addition to model (13). We compared the performance between models (13) and (14) for turn-changing prediction. Model (14) had a significantly higher F1 score, 0.797, than model (13), 0.771 (*p* < 0.001). This suggests that multi-task learning incorporating turn-management willingness prediction into turn-changing predictive models improves the performance of turn-changing prediction.

We also analyzed whether multi-task learning is useful for predicting turn-management willingness. We compared the performance between models (13) and (14). Model (14) had significantly higher CCCs, 0.580 for turn-holding, 0.530 for turn-yielding, and 0.560 for listening, than model (13), 0.556 for turn-holding, 0.504 for turn-yielding, and 0.549 for listening (*p* < 0.05). This suggests that multi-task learning also improved the performance of the speaker's turn-management willingness prediction only when using the features of speakers and listeners.

## 7. Discussion

### 7.1. Answer to Q1

In Section 4, we observed dissonance between the willingness score and actual next speaker in turn-changing. In detail, we found that many turn-changes happened even when the speaker had a high turn-holding willingness to continue speaking and the listener had a low turn-grabbing willingness to continue listening. This means that there are discrepancies between willingness and actual speaking behavior (i.e., turn-changing). This dissonance between willingness and actual speaking behavior during turn-changing is the first time that such a dissonance has been revealed. Previous studies have focused primarily on what verbal and non-verbal behaviors humans engage in during turn-changing. In the future, it may be possible to examine how verbal and non-verbal behaviors change depending on the type of participant's willingness during turn-changing.

The results also suggest that there is a possibility that willingness prediction could be beneficial for building an agent that has smooth turn-management based on the discrepancies between willingness and actual turn-changing. For example, the agent may be able to prompt a listener to take a turn and start speaking by exhibiting verbal and nonverbal behavior.

The willingness we collected was not reported by the individuals participating in the dialogue but by a third party observing the video. The agreement rate of the willingness data annotated by the 10 outside observers was very high and considered to be of high quality. However, it is not certain that judgments on willingness by third parties and the participants themselves will be exactly the same. It would be desirable to examine what differences exist when participants themselves and third parties annotate willingness. The data we used was dialogue data between two Japanese in a discussion featuring divergent opinions. It is conceivable that the topic, number of people, culture, language, and other conditions could have a variety of effects on speaker alternation. It would also be interesting to examine how these conditions affect the relationship between willingness and actual turn-changing.

### 7.2. Answer to Q2-1

Our results show that the listener's acoustic information is most useful for predicting the speaking and listening willingness of speakers and listeners when using only a single feature from speakers or listeners. Generally, the listener uses short bits of speech and non-verbal behaviors such as nodding, changing the head direction, and gazing (Duncan, 1972). Our results suggest that the listener's acoustic backchannel has the potential to be the most useful for indicating the listener's willingness to speak and listen. Moreover, this is a very interesting result since the acoustic features of the listener are most useful for predicting not only the listener's willingness but also the speaker's willingness. Thus, the listener's reaction influences the speaker's willingness (to continue speaking or to stop and listen) heavily. A speaker's linguistic features is most useful for predicting turn-changing when features from either the speaker or listener are used. One explanation is that the speaker's verbal behavior is one of the most useful cues for yielding and holding a turn (Kendon, 1967). This is a very interesting result since the most useful modality differs between willingness prediction and turn-changing prediction.

Moreover, our results show that the features of both speakers and listeners are useful for predicting turn-management willingness. Individual turn-management willingness can be predicted better using features from individuals rather than from others. Individual willingness is well reflected in an individual's behavior. Moreover, the models using features of both speakers and listeners performed better than those using only speaker or listener features. When using features of both the speaker and listener, the acoustic modality is the best-performing modality for predicting the speaking and listening willingness among the trimodal features. One explanation is that the listener's acoustic information is most useful for predicting speaking willingness when using a single feature from a single modality. Moreover, the multimodal approach with the trimodal features of the speaker and listener is most useful in predicting the turn-management willingness of both persons. In other words, the turn-management willingness of a speaker and listener can influence the verbal and non-verbal behaviors of both. This suggests that predicting the internal state of an individual, such as their willingness, using features from not only the individual but also conversational partners could be greatly useful in dyad interactions.

### 7.3. Answer to Q2-2

Turn-changing prediction becomes most accurate when turn-management willingness and turn-changing are predicted simultaneously using multi-task learning. This demonstrates that explicitly adding willingness as a prediction target improves the performance of turn-changing prediction. This introduces new possibilities for more accurately predicting human behavior by predicting human psychological states at the same time in conversations. Moreover, models that jointly learn two tasks also improve the performance of turn-management willingness compared with models that perform just one task. The multi-task learning approach allows a model to learn the underlying relationship between willingness scores and turn-changing. This results in both improved turn-changing and turn-management willingness prediction. These results also suggest that a multi-task prediction approach that predicts the internal state of people, such as their willingness and actual behaviors, could be greatly useful in dyad interactions. Applying such an approach to tasks other than turn-changing prediction will be part of our further investigation.

### 7.4. Future work

For conversational agents or robots to start or stop speaking at the right time, we do believe that predicting human turn-management willingness is critically important, rather than simply predicting the next speaker (actual turn-changing). In this study, we attempted to predict the willingness of two conversation partners simultaneously during dyad interactions. When considering a human-agent interaction (HAI) scenario, our approach would need to be adapted to predict only one user's willingness using the tri-modal features, for either a speaker role or a listener role. This is one of our future research directions.

Modeling turn-management willingness may aid in detecting discrepancies between willingness toward turn-changing and actual turn-changing. A conversational system can then recognize users having a high willingness to speak (speaker's turn-holding or listener's turn-grabbing willingness) even though they cannot speak. It could even help to mediate meetings by possibly interrupting the current speaker if a person does not notice that the conversation partner has a low willingness to listen. Many studies have been conducted to facilitate human interactions with agents and robots. For example, robots have been proposed to prompt the user who has the least dominance in a conversation (Nakano et al., 2015). With such facilitation, the appropriate time at which an agent can prompt a user to speak could be recognized with our prediction results on turn-management willingness and turn-changing.

Our goal is to study turn-management willingness and its impact on turn-changing precision. We used high-level abstracted features extracted from acoustic, linguistic, and visual modalities. We plan to use other interpretable features, such as prosody (Ferrer et al., 2002; Holler and Kendrick, 2015; Hömke et al., 2017; Holler et al., 2018; Masumura et al., 2018, 2019; Roddy et al., 2018) and gaze behavior (Chen and Harper, 2009; Kawahara et al., 2012; Jokinen et al., 2013; Ishii et al., 2015a, 2016a) and to implement more complex predictive models (Masumura et al., 2018, 2019; Roddy et al., 2018; Ward et al., 2018) that take into account temporal dependencies.

Hara et al. (2018) proposed a predictive model that can predict backchannels and fillers in addition to turn-changing using multi-task learning. To analyze and model the relationship between turn-management willingness, backchannels, and fillers would be interesting future work.

We also plan to incorporate predictive models into conversational agent systems that can leverage smooth turn-changing and facilitate the start of speaking for those who cannot speak despite having a high turn-holding or turn-grabbing willingness.

## 8. Conclusion

We found that many turn-changes happen even when the speaker has a high turn-holding willingness to continue speaking and the listener has a low turn-grabbing willingness to continue listening. This means that there are discrepancies between willingness and actual speaking behavior (i.e., turn-changing). Conversational agents could perform smooth turn-changing and facilitate users in speaking with the prediction results of turn-management willingness and actual turn-changing. We built models for predicting the turn-management willingness of speakers and listeners as well as turn-changing with trimodal behaviors, acoustic, linguistic, and visual cues, in conversations. An evaluation of our models showed that turn-management willingness and turn-changing are predicted most precisely when all of the modalities from speakers and listeners are used. Furthermore, turn-changing prediction becomes more accurate when turn-management willingness and turn-changing are predicted jointly using multi-task learning. Turn-management willingness prediction also becomes more accurate with combined prediction. These results suggest that more accurate predictive models of human behaviors could be built by incorporating other predictions related to human psychological states.

## Data availability statement

The original contributions presented in the study are included in the article/supplementary files, further inquiries can be directed to the corresponding authors.

## Ethics statement

The studies involving human participants were reviewed and approved by the Ethical Review Committee at NTT Corporation. The patients/participants provided their written informed consent to participate in this study.

## Author contributions

RI and L-PM contributed to conception and design of the study. RI organized the corpus and wrote the first draft of the manuscript. XR performed implementation and experiment of prediction model. XR and MM performed the statistical analysis. RI, XR, MM, and L-PM wrote sections of the manuscript. All authors contributed to manuscript revision, read, and approved the submitted version.

## Funding

XR and L-PM were partially supported by the National Science Foundation (#1722822 and #1734868). MM was supported by the Swiss National Science Foundation (#P2GEP2_184518). RI was supported by the NTT Corporation. The funder was not involved in the study design, collection, analysis, interpretation of data, the writing of this article or the decision to submit it for publication.

## Conflict of interest

Author RI was employed by NTT Corporation. The author was a visiting researcher at CMU and conducted this research as a research fellow at CMU. The remaining authors declare that the research was conducted in the absence of any commercial or financial relationships that could be construed as a potential conflict of interest.

## Publisher's note

All claims expressed in this article are solely those of the authors and do not necessarily represent those of their affiliated organizations, or those of the publisher, the editors and the reviewers. Any product that may be evaluated in this article, or claim that may be made by its manufacturer, is not guaranteed or endorsed by the publisher.

## Footnotes

^1^http://nlp.ist.i.kyoto-u.ac.jp/index.php?BERT%E6%97%A5%E6%9C%AC%E8%AA%9EPretrained%E3%83%A2%E3%83%87%E3%83%AB.
